# Functional Metaplasticity of Hippocampal Schaffer Collateral-CA1 Synapses Is Reversed in Chronically Epileptic Rats

**DOI:** 10.1155/2017/8087401

**Published:** 2017-10-02

**Authors:** Mirko Rehberg, Timo Kirschstein, Xiati Guli, Steffen Müller, Marco Rohde, Denise Franz, Tursonjan Tokay, Rüdiger Köhling

**Affiliations:** ^1^Oscar Langendorff Institute of Physiology, University of Rostock, Rostock, Germany; ^2^School of Science and Technology, Nazarbayev University, 53 Kabanbay batyr ave., Astana 010000, Kazakhstan

## Abstract

Spatial learning and associating spatial information with individual experience are crucial for rodents and higher mammals. Hence, studying the cellular and molecular cascades involved in the key mechanism of information storage in the brain, synaptic plasticity, has led to enormous knowledge in this field. A major open question applies to the interdependence between synaptic plasticity and its behavioral correlates. In this context, it has become clear that behavioral aspects may impact subsequent synaptic plasticity, a phenomenon termed behavioral metaplasticity. Here, we trained control and pilocarpine-treated chronically epileptic rats of two different age groups (adolescent and adult) in a spatial memory task and subsequently tested long-term potentiation (LTP) in vitro at Schaffer collateral—CA1 synapses. As expected, memory acquisition in the behavioral task was significantly impaired both in pilocarpine-treated animals and in adult controls. Accordingly, these groups, without being tested in the behavioral training task, showed reduced CA1-LTP levels compared to untrained young controls. Spatial memory training significantly reduced subsequent CA1-LTP in vitro in the adolescent control group yet enhanced CA1-LTP in the adult pilocarpine-treated group. Such training in the adolescent pilocarpine-treated and adult control groups resulted in intermediate changes. Our study demonstrates age-dependent functional metaplasticity following a spatial memory training task and its reversal under pathological conditions.

## 1. Introduction

Spatial learning and the ability to associate spatial information with individual experience are crucial in the habitat of rodents and higher mammals. Consequently, the cellular basis of information storage in the brain is one of the most intensively studied issues in neurophysiology, and it is widely accepted that the hippocampus plays an instrumental role for rodent spatial learning and memory.

On the one hand, the hippocampus harbors neurons that are specifically active when the animal crosses a certain place. The first evidence for the existence of these so-called place cells [1] was given by O'Keefe and Dostrovsky [2] and largely confirmed by many other groups [3]. Although it became obvious that the whole medial temporal lobe and other brain areas also contribute to spatial learning [4], the hippocampus is still regarded as a major brain structure involved in spatial orientation and memory.

On the other hand, hippocampal synapses show a tremendous propensity to increase their strength to prior intense neuronal activity, known as long-term potentiation (LTP) [5]. The idea that hippocampal LTP is in fact the cellular key mechanism of spatial memory formation is largely based on studies demonstrating the concordance of impaired spatial learning behavior and reduced LTP, particularly at the Schaffer collateral-CA1 synapse (reviewed in [6]). Only a few reports have directly linked behavior and in vivo LTP [7, 8]. With respect to the question of whether LTP and behavioral learning share common cellular mechanisms, it is intriguing to study the experience dependence of synaptic plasticity [9] or “metaplasticity” [10] occurring after behavioral learning and memory acquisition. Metaplasticity can be induced by electrical stimulation in vivo [11]. Furthermore, there is a large body of evidence suggesting that behavioral stress impairs CA1-LTP in vitro [12, 13] and in vivo [14, 15]. Moreover, behavioral stress was also shown to facilitate CA1-LTD (long-term depression) in vitro [13] and in vivo [15]. Such behavioral stress included restraint and tailshocks, all of which is aversive and not related to formal learning paradigms. In contrast to data on behavioral stressors, there are only few reports on “behavioral metaplasticity” [16] following the acquisition of spatial information in vivo, which has, after habituation to the Morris water maze, no aversive situation [17]. At least the counterpart of LTP, long-term depression (LTD), in freely moving animals was sensitive to explorative behavior in a novel environment [18]. Further, stress-induced impairment of corticostriatal LTP in healthy adult hamsters was reversed under dystonic conditions [19]. It is, thus, important to answer the question whether in vivo training of spatial memory alters subsequent LTP in vitro and whether intense neuronal activity occurring during status epilepticus modifies such in vivo plasticity changes. To test this, we trained control and pilocarpine-treated chronically epileptic rats in the Morris water maze hidden platform task and subsequently recorded CA1-LTP in hippocampal slice preparations after completing seven days of spatial memory formation.

## 2. Materials and Methods

### 2.1. Animal Groups

In this study, two different age groups of Wistar rats (Charles River, Sulzfeld, Germany) were studied. In the adolescent animal group, training was performed in 10–14 week-old rats, followed by electrophysiological recordings of brain slices at 11–15 weeks of age (*n* = 10 control and 5 epileptic rats). In contrast, in the adult animal group, training was performed in 24–27 week-old rats and electrophysiological recordings followed at 25–28 weeks of age (*n* = 9 control and 9 epileptic rats). In all cases, electrophysiological recordings were carried out one day after completing the spatial memory training period. Electrophysiological data from trained rats were compared to electrophysiological data from untrained rats (adolescent: *n* = 14 control and 7 epileptic rats; adult: *n* = 6 control and 6 epileptic rats).

### 2.2. Status Epilepticus and Chronically Epileptic Animals

**Figure 1:**
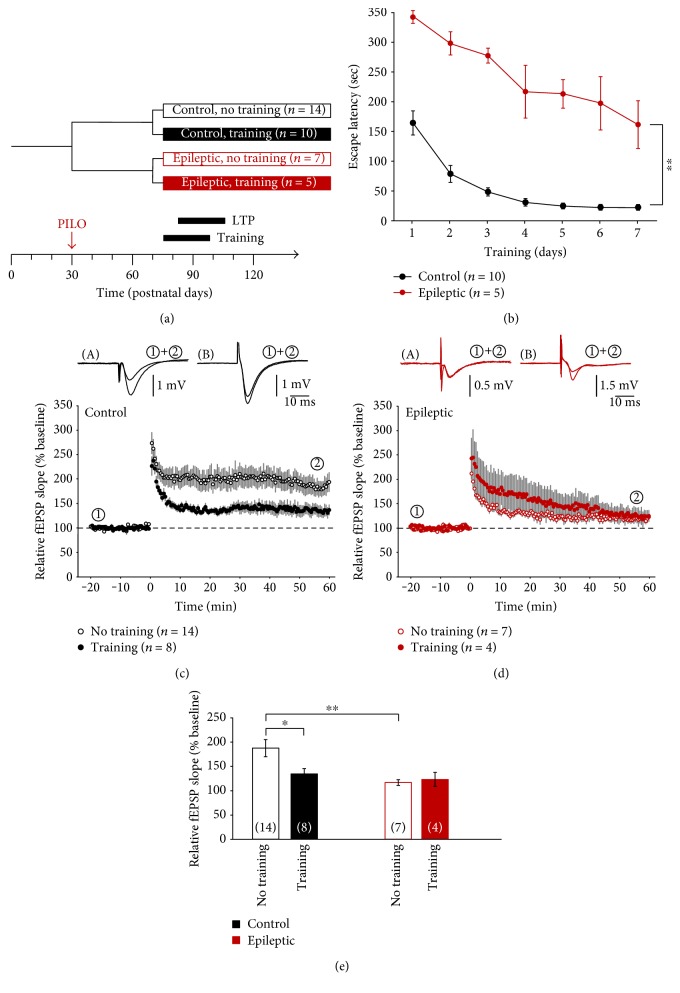
Functional metaplasticity by spatial memory training in adolescent rats. (a) Experimental paradigm indicating pilocarpine-induced status epilepticus (PILO) and time points of both training and LTP (which was always tested one day after completing seven days of training). Four groups of animals were tested (control and epileptic rats, with or without training). (b) Spatial memory acquisition (as assessed by the cumulative escape latency of six consecutive trials per day) was significantly impaired in epileptic animals (red symbols) compared to controls (black symbols). (c, d) Spatial memory training (filled symbols) significantly reduced subsequent CA1-LTP in vitro in controls as compared to untrained controls (open symbols) but had no significant effect on CA1-LTP in epileptic animals. (e) Bar graphs summarizing CA1-LTP levels of the four groups of animals. ^∗^*P* < 0.05 and ^∗∗^*P* < 0.01.

**Figure 2:**
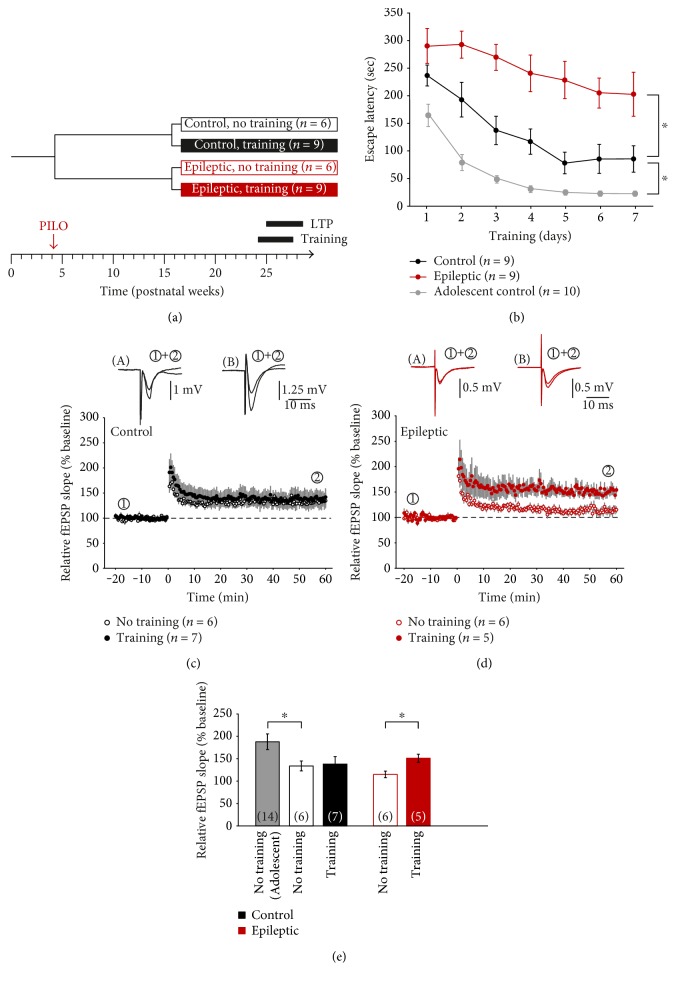
Functional metaplasticity by spatial memory training in young adult rats. (a) Experimental paradigm indicating pilocarpine-induced status epilepticus (“PILO”) and time points of both training and LTP in four groups of animals (control and epileptic rats, with or without training). (b) Spatial memory acquisition was again significantly impaired in epileptic animals (red symbols) compared to controls (black symbols). Moreover, there was also a significant difference between adolescent (gray symbols, data taken from Figure 1(b) for the sake of clarity) and young adult control animals (black symbols). (c, d) Spatial memory training (filled symbols) had no effect on subsequent CA1-LTP in vitro in controls but significantly enhanced CA1-LTP in young adult epileptic animals. (e) Bar graphs summarizing CA1-LTP levels of the four groups of animals. In addition, LTP data from adolescent controls taken from Figure 1(e) is given for comparison. ^∗^*P* < 0.05.

Half of the animals were treated with pilocarpine hydrochloride (340 mg/kg, i.p.) at the age of 30 days in order to induce status epilepticus (PILO, Figures 1(a) and 2(a)). The protocol using pilocarpine hydrochloride is described elsewhere [20–22]. Briefly, rats were given methyl-scopolamine nitrate (1 mg/kg, i.p.) 30 min prior to pilocarpine treatment in order to reduce peripheral cholinergic effects. Then, pilocarpine hydrochloride or saline (referred to as control animals) was applied, and the animals were carefully monitored to determine the onset of status epilepticus which was terminated after 40 min by injection of diazepam solution (Ratiopharm, Ulm, Germany; 10 mg/kg, i.p.). Occasionally, diazepam had to be reinjected in order to stop seizure activity. When status epilepticus did not develop within 60 min, rats were given a second pilocarpine dose (170 mg/kg, i.p.). Finally, the rats were fed with 5% glucose solution for 1 day and kept in separate cages. All procedures were performed according to national and international guidelines on the ethical use of animals (European Council Directive 86/609/EEC, approval of local authority LALLF M-V/TSD/7221.3-1.1-004/06). All efforts were made to minimize animal suffering and to reduce the number of animals used.

Animals that have been treated with PILO start to present with unprovoked seizures 2-3 weeks after status epilepticus with 4–6 seizures per day, lasting on average 42 s [23, 24]. In long-term EEG recordings, epileptic rats show about 40 spike periods per day with a mean duration of 6 min [24].

### 2.3. Spatial Memory Training

Behavioral experiments were performed in order to train spatial memory using the classical Morris water maze hidden platform task [20]. Briefly, a circular platform (diameter 15 cm, 1-2 cm below the water surface) was randomized to one of four quadrants of the water maze (110 cm diameter) for each animal; for a given animal, the platform position remained fixed. The animal was randomly placed in the maze (one of eight different positions) and was allowed to search 60 s to reach the platform. When the animal failed to find the platform within 60 s, it was manually placed onto the platform. Once on the platform, the animal was allowed to rest for 30 s in order to view large-scale cues on all four walls around the water maze, followed by another 60 s resting time in the cage before the next trial was started (6 consecutive trials per day, always different insertion positions). Water and room temperatures were monitored, and the animal's movement in the maze was filmed and recorded by a tracking software (Ethovision Color, Noldus, The Netherlands) analyzing the latency to reach the platform. Spatial memory training was performed during one period of seven consecutive days.

### 2.4. Slice Preparation and Maintenance

Hippocampal slices were prepared one day after the last trial in the Morris water maze (i.e., 12–24 h after the last trial). After deep anesthesia with diethyl ether, rats were decapitated and the brain was rapidly removed and submerged into oxygenated ice-cold dissection solution containing (in mM) 125 NaCl, 26 NaHCO_3_, 3 KCl, 1.25 NaH_2_PO_4_, 0.2 CaCl_2_, 5 MgCl_2_, and 13 D-glucose (95% O_2_, 5% CO_2_; pH 7.4; 306–314 mosmol/kg). Horizontal brain slices (400 *μ*m) of the hippocampus were prepared using a vibratome (Campden Instruments, Loughborough, UK), and slices were then transferred into a holding chamber containing artificial cerebrospinal fluid (ACSF) containing (in mM) 125 NaCl, 26 NaHCO_3_, 3 KCl, 1.25 NaH_2_PO_4_, 2.5 CaCl_2_, 1.3 MgCl_2_, and 13 D-glucose (306–314 mosmol/kg). Slices were continuously bubbled with 95% O_2_ and 5% CO_2_ to maintain the pH at 7.4 and were allowed to recover at room temperature (20–22°C) for at least 1 hour before being transferred into recording chamber.

### 2.5. Induction of Long-Term Potentiation

Hippocampal slices were transferred into an interface chamber and continuously superfused with oxygenated ACSF at a flow rate of 2 ml/min with a volumetric infusion pump MCM-500 (MC Medicine technique GmbH, Alzenau, Germany), and the solution temperature was controlled at 32 ± 1°C (npi electronic GmbH, Tamm, Germany). The experiments started after an equilibration time of at least 30 min. Field excitatory postsynaptic potentials (fEPSP) were recorded using borosilicate glass pipettes (2-3 MΩ, pulled with PIP5 from HEKA Electronik, Lambrecht, Germany) filled with ACSF. Stimulating and recording electrodes were placed into CA1 stratum radiatum in order to study the Schaffer collateral-CA1 synapse. Bipolar stimulation was performed with platinum wire electrodes and applied to Schaffer collaterals with an ISO-STIM01M stimulus isolator (npi electronic GmbH, Tamm, Germany). The afferent fibers were stimulated at a rate of 0.033 Hz with the baseline stimulation strength adjusted to 30–40% of the maximal fEPSP amplitude. After a stable baseline recording for at least 20 min was achieved, a high-frequency stimulation (HFS) paradigm consisting of 100 pulses at 100 Hz at double baseline stimulation intensity was delivered in order to induce long-term potentiation (LTP) using a Master-8 stimulator (A.M.P.I., Jerusalem, Israel).

Recording signals were amplified and filtered at 1 kHz by an EXT-10-2F (npi electronic GmbH, Tamm, Germany). Analog data were digitized with a Micro1401 analog-to-digital converter (Cambridge Electronic Design, Cambridge, UK) and stored for off-line analysis using Signal 2.16 software (Cambridge Electronic Design, Cambridge, UK). All chemicals used for physiological solutions were purchased from Sigma-Aldrich (Taufkirchen, Germany).

Baseline fEPSP slopes were measured as a mean of twenty consecutive frames (i.e., 10 min) just before LTP induction (min −10 to 0, “baseline”), and LTP was assessed at the end of the experiment by averaging ten consecutive frames (min 55–60, “LTP”). Electrophysiological data from all slices of a given animal were averaged to obtain a single “animal LTP” time course, and then all “animal LTP” time courses were averaged to a mean “group LTP” time course.

### 2.6. Statistical Analysis

All data are expressed as mean values ± the standard error of the mean (SEM). Statistical comparisons of behavioral as well as electrophysiological data were performed between the groups of rats using the two-way ANOVA (spatial memory training) and the Mann–Whitney *U* test (LTP data) with the level of significance set to *P* < 0.05.

## 3. Results

Since we aimed to investigate functional metaplasticity in the hippocampal CA1 area in control and chronically epileptic animals, four experimental groups were assigned (Figure 1(a)). Following pilocarpine-induced status epilepticus at postnatal day 30 (arrow “PILO” in Figure 1(a)), each group of animals (“control” or “epileptic”) was further divided into a group that received a period of spatial memory training of one week (filled bars) which was performed at 10–14 weeks of age and another group of animals that received no training (open bars). Spatial memory assessed by the cumulative escape latency of six consecutive trials per day was severely impaired in pilocarpine-treated rats (red symbols) as compared to control animals (black symbols, Figure 1(b); *P* < 0.01). One day after completing the spatial memory task, hippocampal brain slices were prepared and CA1 long-term potentiation (LTP) was induced by high-frequency stimulation (HFS, time point “0,” in Figures 1(c) and 1(d)). In line with the spatial memory data, slices from untrained adolescent control rats exhibited significantly more LTP (188 ± 18% of baseline, *n* = 23 slices from 14 animals) than slices from untrained pilocarpine-treated animals (117 ± 6%, *n* = 21 slices from 7 animals, *P* < 0.01; Figure 1(e)). Moreover, as expected from stress-induced metaplasticity, slices from trained control rats showed significantly less LTP as compared to untrained controls (134 ± 11%, *n* = 16 slices from 8 animals, *P* < 0.05; Figure 1(c)). In marked contrast, slices from trained pilocarpine-treated rats in this age group (123 ± 14%, *n* = 11 slices from 4 animals) did not differ from LTP levels observed in untrained epileptic animals (Figure 1(d), summarized in Figure 1(e)). Thus, spatial memory training in vivo reduced subsequent CA1-LTP in adolescent controls but had no significant effect on subsequent CA1-LTP in epileptic rats of this age.

Next, we asked whether this discrepancy holds on during aging and repeated these experiments with rats that received pilocarpine at postnatal day 30 but were analyzed at 24–28 weeks of age (Figure 2(a)). In this adult animal group, spatial learning was again impaired in pilocarpine-treated animals (red symbols, Figure 2(b); *P* < 0.05) as compared to adult controls (black symbols), but the latter group showed also significantly poorer performance compared to adolescent controls (gray symbols, data from Figure 1(b); *P* < 0.05). Consistent with the age-related impairment of spatial memory formation, LTP in slices from untrained adult controls (133 ± 11%, *n* = 16 slices from 6 animals) was also significantly reduced compared to LTP in slices from adolescent controls (*P* < 0.05 versus young controls; Figures 2(c) and 2(e)). As in the young age group, slices from untrained adult pilocarpine-treated rats showed less LTP than untrained adult controls, but this difference was not significant (115 ± 7%, *n* = 18 slices from 6 animals; Figure 2(d)). However, spatial memory training had significant LTP-enhancing effects in pilocarpine-treated rats (152 ± 9%, *n* = 13 slices from 5 animals; *P* < 0.05; Figures 2(d) and 2(e)), but not in controls (138 ± 16, *n* = 17 slices from 7 animals).

## 4. Discussion

In the present study, we found that both aging and chronic epilepsy were associated with impaired spatial memory performance in the Morris water maze task with concomitantly compromised CA1-LTP. Thus, alterations that occur during aging or after status epilepticus appear to jointly impact both CA1-LTP and spatial memory. The concordant impairment of spatial memory and CA1-LTP is largely consistent with numerous reports (reviewed by [6]) and further supports the idea that CA1-LTP is a major cellular mechanism involved in spatial memory.

An even more important result of our study was the significant functional metaplasticity in control animals with reduced CA1-LTP following spatial memory training. Besides the well-known inhibitory effect of behavioral stress on in vitro CA1-LTP [12, 25, 26], some reports are available that studied more complex in vivo effects, namely, enriched environment, on subsequent synaptic plasticity in vitro [27, 28]. The present experiments add new information to the concept of behaviorally evoked changes in synaptic plasticity, because spatial memory training also dampens the propensity of subsequent in vitro CA1-LTP in adolescent rats. In contrast to controls, in pilocarpine-treated adolescent animals, spatial memory training had no effect on CA1-LTP. This is an interesting finding since pilocarpine-treated animals suffered from impaired spatial learning, and this argument may indicate that significant functional metaplasticity may only be expected if spatial memory acquisition is preserved. Alternatively, there is no behavioral metaplasticity in adolescent epileptic rats since no significant LTP was observed in these rats. On the other hand, slices from adult pilocarpine-treated animals showed significantly enhanced CA1-LTP as compared to untrained rats. Therefore, the question arises whether successful memory acquisition is really necessary for metaplasticity of CA1-LTP. Alternatively, it is conceivable that only the amount of physical activity, which may be regarded as behavioral stress [12, 24, 25], could have led to the LTP-reducing effect of the behavioral task.

Hence, what are the determinants of behavioral metaplasticity? First, functional metaplasticity clearly depends on age. This is a common finding in metaplasticity of in vitro slice preparations [29, 30]. Thus, the sensitivity of synapses towards metaplastic challenges is changing during development and the same conditioning paradigm might have different effects on the same synapse later in life. While there is no doubt of cognitive decline in humans [31, 32] which is generally ascribed to atrophy in temporal and extratemporal brain structures [33, 34], the mechanisms involved in reduced LTP in adult animals are less understood. There is some evidence suggesting a shift in LTP threshold [35–37], which was partly explained by a decreased activity of serine racemase following oxidative stress [38–40]. Interestingly, CA1 minislices from pilocarpine-treated rats showed decreased expression of serine racemase and amino acid transporter Asc-1 but enhanced expression of D-amino oxidase and Grin2B levels [20, 41] suggesting D-serine dysfunction in the CA1 area of pilocarpine-treated animals. With respect to spatial learning and functional metaplasticity, it is hence an attractive working hypothesis that hippocampal degeneration and predominantly CA1 neuron loss [21, 42, 43] following pilocarpine-induced status epilepticus may mimic the situation in healthy adult animals. Along this argument, both epilepsy and age can impair learning performance, but the slopes of the learning curves between adolescent and adult animals were quite similar; this may indicate that spatial learning in adult animals recruited extrahippocampal circuits. Although this question cannot be answered in the present study, this issue is worth to be addressed in the future.

Furthermore, age played also an important role in stress-induced in vivo metaplasticity of corticostriatal LTP in healthy hamsters [19] and, again, increased Grin2B levels were observed to be involved in the difference between control and dystonic hamsters [44]. An attractive alternative explanation to stress-induced metaplasticity, however, is the possibility that synaptic plasticity was partially occluded by prior acquired memory [7]. Furthermore, experimentally evoked LTP at the hippocampal CA3-CA1 synapse blocked the acquisition of hippocampal-dependent learning tasks [8]. Whether stress or rather memory acquisition was the major determinant for LTP impairment in trained animals, age-dependent changes in NMDA receptor function seem to be a major determinant for different forms of metaplasticity.

In addition, metaplasticity described here also depends substantially on the paradigm used for the induction of synaptic plasticity. In the present study, high-frequency stimulation-induced LTP was significantly reduced in the epileptic CA1 area, but an earlier study has found enhanced CA1-LTP induced by theta burst stimulation in the same tissue, albeit at a younger age [41]. Even more importantly, HFS-induced LTP in that study showed a substantially higher magnitude and did not differ between control and pilocarpine-treated animals [41]. Since animals that had experienced status epilepticus suffer from chronic epilepsy, altered levels of CA1-LTP in this tissue can be regarded as functional metaplasticity following behavioral seizures. Thus, the different effects of behavioral seizures on CA1-LTP suggest that not only age but also induction mechanisms of synaptic plasticity are major determinants for both the direction and the magnitude of metaplasticity.

What is the relevance of such a form of metaplasticity? As already proposed by early seminal papers, metaplasticity serves as a homeostatic function preventing saturation of synaptic plasticity [9, 10]. In this context, functional metaplasticity appears to be an unequivocal prerequisite for the idea that learning behavior and synaptic plasticity share common cellular mechanisms. Importantly, since enriched environment exerts significant influence on subsequent synaptic plasticity in vitro [27, 28], there is also evidence for favorable effects of physical exercise on both in vivo seizure rates and in vitro hyperexcitability [45–47], which were also translated to epilepsy patients [45].

The present study demonstrates functional metaplasticity following a spatial memory training task which is in line with previous observations of stress-induced LTP reduction but also adds novel information about metaplasticity at different ages and under pathological conditions such as chronic epilepsy. While in young control animals LTP levels are dampened following spatial memory training, the same training facilitates LTP in older pilocarpine-treated rats suggesting that spatial learning may act as a homeostatic regulator of synaptic plasticity to an intermediate and unsaturated level in order to preserve further adaptations of synaptic strength.
